# Development of a Genus-Specific *Brucella* Real-Time PCR Assay Targeting the 16S-23S rDNA Internal Transcribed Spacer from Different Specimen Types

**DOI:** 10.3390/vetsci7040175

**Published:** 2020-11-11

**Authors:** Rejoice Nyarku, Ayesha Hassim, Annelize Jonker, Melvyn Quan

**Affiliations:** Vectors and Vector-borne Diseases Research Programme, Department of Veterinary Tropical Diseases, Faculty of Veterinary Science, University of Pretoria, Private Bag X04, Onderstepoort 0110, South Africa; esenyarku@gmail.com (R.N.); ayesha.hassim@gmail.com (A.H.); annelize.jonker@up.ac.za (A.J.)

**Keywords:** diagnosis, qPCR, brucellosis, blood, milk, abomasal fluid

## Abstract

The aim of this study was to develop a 16S-23S ribosomal deoxyribonucleic acid internal transcribed spacer (ITS) quantitative polymerase chain reaction (qPCR) assay for the early diagnosis and rapid screening of brucellosis. Blood, milk, and tissue samples were spiked with *B. abortus* biovar 1 (B01988-18 strain) to determine the analytical sensitivity and specificity of the assay. The 95% limit of detection of the ITS qPCR assay was highest in tissue, followed by blood, then milk, i.e., 0.48, 4.43, and 15.18 bacteria/PCR reaction, respectively. The diagnostic performance of the assay was compared to the *Brucella* cell surface protein (BCSP) 31 qPCR assay and bacterial culture. Out of 56 aborted foetal tissue samples from bovine, ovine, and caprine, 33% (19/56) were positive for *Brucella* spp. The sensitivity and specificity of the ITS qPCR assay was 87% and 95% respectively, compared to 92% and 89% for the BCSP31 qPCR assay and 47% and 55% for bacterial culture, respectively. The assay was efficient, sensitive, and specific, making it a valuable tool in the early detection of the *Brucella* pathogen.

## 1. Introduction

Brucellosis is a zoonotic disease that causes abortion and infertility in animals and undulant fever in humans [1]. It is distributed worldwide and endemic in developing countries. It is caused by the genus *Brucella* [2] and belongs to the order alpha-Proteobacteria, which consists of mostly intra-cellular bacteria that are recognised as pathogenic in a number of mammalian hosts [3]. Twelve *Brucella* species have been characterised: six classical species, which includes *Brucella suis*, *B. melitensis*, *B. abortus*, *B. ovis*, *B. neotomae*, and *B. canis*; an additional six novel species, which includes *B. ceti* from cetaceans, *B. pinnipedialis* from seals [4], *B. microti* from wild rodents, *B. inopinata* from breast implant infection in humans [5], *B. vulpis* from red foxes [6], and *B. papionis* from baboons [7]. These are classified based on distinction between host preference and pathogenicity [8]. The smooth strains (*B. suis*, *B. melitensis*, and *B. abortus*) are all zoonotic, and of the rough strains (*B. ovis* and *B. canis*), only *B. canis* is zoonotic [9].

Brucellosis is a controlled and notifiable disease in humans and animals across sub-Saharan Africa [10]. Clinical signs vary in animals depending on the host species [11]. These include spontaneous abortion, weak offspring, pyrexia, hygromas and mastitis [12], orchitis, epididymitis, ampullitis, and seminal vesiculitis [13]. However, these clinical presentations are non-specific, making diagnosis difficult. In addition, latent asymptomatic heifers introduced into a naïve herd cause abortion storms. The placenta, foetal fluid, and aborted foetus serve as major sources of infection to other animals [14].

The diagnosis of the disease is by routine serological tests and bacterial culture isolation, which is the “gold standard” in brucellosis diagnosis [15]. However, these tests have inadequate sensitivity and specificity, are time-consuming, and have the potential of causing infection in laboratory personnel. To overcome these challenges, several conventional and real-time PCR assays utilising different primers derived from different polymorphic regions in the *Brucella* genome have been developed [16,17]. While these assays generally work well, immunological cross reactions with other closely related species such as *Ochrobactrum anthropi* have been reported in conventional PCR assays [18], resulting in reduced assay specificity [19]. Some of these assays are only able to differentiate a limited number of species [20]. There is a need to develop accurate and rapid diagnostic assays for high-throughput detection of *Brucella*, in order to support appropriate control measures and contribute to the eradication of the disease in a country.

The 16S-23S ribosomal deoxyribonucleic acid (rDNA) internal transcribed spacer region (ITS) PCR has proved to be a promising diagnostic tool. The 16S-23S ITS has also been used in the diagnosis of brucellosis in humans [21]. It has been proven to be rapid, specific, and more sensitive for the diagnosis of brucellosis in both animals and humans because the rDNA exists in multiple copies [22]. The ITS region is highly conserved, with 100% sequence homology among *Brucella* spp. [23].

The aim of this study was to develop a rapid genus-specific qPCR assay to detect *Brucella* species targeting the 16S-23S rDNA ITS region.

## 2. Materials and Methods

### 2.1. Assay Design

Using *B. abortus* (GenBank accession number of X95889) as a template, nucleic acid sequences of the 16S-23S rDNA ITS region of *Brucella* spp. were retrieved from GenBank [24], using the nucleotide Basic Local Alignment Search Tool (BLAST^®^, National Center for Biotechnology Information, Bethesda, MD, USA) [24,25], and the highly similar sequences programme (megablast, National Center for Biotechnology Information, Bethesda, MD, USA). An *O. intermedium* sequence (AJ867325) was used as a template, together with the discontiguous megablast programme, to obtain sequences of other bacteria with close homology to *Brucella*.

The retrieved genetic sequences were aligned online using a multiple sequence alignment programme (MAFFT version 7) [26]. Data analysis in molecular biology and evolution software (DAMBE) [27] was used to identify and remove identical sequences and sequences were edited with BioEdit [28]. A TaqMan^®^ minor groove binder (MGB) assay was designed using Primer Express v3.0 using default primer/probe design parameters: melting temperature (Tm) of 58–60 °C and 68–70 °C respectively, a GC% content of 30–80%, the last five nucleotides of the 3′ end of the primer do not contain more than two ‘G’ and ‘C’ residues, and the 5′ end of the probe does not contain a ‘G’ residue (ThermoFisher Scientific, Waltham, MA, USA). The amplicon length was 72 bp. 

Field strains of *Brucella* spp. obtained from the Department of Veterinary Tropical Disease (DVTD) diagnostics laboratory, University of Pretoria (UP) and closely related non-*Brucella* species, such as *Ochrobactrum anthropi* were used for this study (Table 1). All bacterial isolates were sub-cultured on blood agar at 37 °C for 72 h for *Brucella* spp. and 24 h for other bacteria [22]. *Chlamydia abortus* was cultured on a liquid yeast based medium, serum casein glucose yeast extract medium recommended by the supplier of the control strain. The host used was *Acanthamoeba castellani*. Two millilitres (mL) of medium and 20–50 microlitres (µL) of *Acanthamoeba castellani* in a plastic 5 mL screw top tube was used. After three days when a white precipitate at the bottom of the tube was seen, 50 µL of *Chlamydia abortus* was added and reincubated in normal air at 30 °C for seven days. 

Cultures were obtained from the DVTD diagnostic laboratory. Colonies were harvested and stored in 10% glycerol and stored at −80 °C for further use. Then, these were thawed, and sub-cultures were prepared. These were streaked and inoculated on Selecta-MEDIA Blood Tryptose agar +5% sheep blood agar (ThermoFisher Scientific, Waltham, MA, USA). The *Brucella* strains were cultured in an HF151UV CO_2_ incubator (Heal Force, Shangai, China) for 72 h. Bacterial colonies on the plates were scraped off using 10 µL sterile inoculating loops and transferred to 1.5 mL Eppendorf tubes containing 200 µL phosphate-buffered saline (PBS) (Sigma-Aldrich, St Louis, MO, USA). Bacteria were quantified using a TC20™ automated cell counter (Bio-Rad Laboratories, Singapore).

### 2.2. Control Samples

A negative blood control sample was collected from a cow with no history of brucellosis. A complete blood count and microscopic examination for haemoparasites was performed on the sample by the Clinical Pathology Laboratory, Onderstepoort Veterinary Teaching Hospital, UP. A negative fresh milk (bulk tank milk) control sample was collected from a farm with no history of brucellosis, located at Irene, Gauteng, South Africa. Negative tissue control samples from the abattoir were collected from bovine, caprine, and ovine. All samples (blood, milk, and tissue) tested negative for *Brucella* spp. by the ITS qPCR assay.

*Brucella* colonies cultured on blood agar medium were scraped off the culture plates using a sterile inoculating loop and harvested into 1 mL of PBS (Sigma-Aldrich, St Louis, MO, USA). This was the stock solution used for all experiments throughout the study. The number of cells/mL of stock solution (*Brucella abortus* bv. 1 (B01988-18 strain) with a bacterial cell count of 3.19 × 10^7^ cells/mL) was determined using a TC20™ automated cell counter (Bio-Rad Laboratories, Hercules, CA, USA). Blood and milk samples (180 µL) were spiked with 20 µL of a stock *B. abortus* solution and 20–30 milligrams (mg) of *Brucella* positive foetal aborted tissue (confirmed by culture isolation) were tested by the ITS qPCR assay and used as positive controls.

### 2.3. Nucleic Acid Purification 

Nucleic acid was purified using the KingFisher™ Duo Prime purification system and the MagMAX CORE™ Nucleic Acid Purification Kit (ThermoFisher Scientific, Waltham, MA, USA) according to the manufacturer’s instructions. Nucleic acid from blood and milk samples (200 µL) was purified using the simple workflow and eluted in 90 µL of elution buffer before storage of the DNA at −20 °C. The simple and digestion methods were compared in purifying DNA from aborted foetal tissues. The latter was determined to be superior, as this method produced lower cycle threshold results with higher fluorescence on qPCR. Proteinase K (PK) solution, composed of (PK Buffer and MagMax CORE Proteinase K) was added to approximately 20–30 mg of tissue and incubated for 2 h at 55 °C. The supernatant was processed as recommended by the manufacturer. The complex workflow was used to purify DNA from 300 µL foetal abomasal fluid.

VetMAX™ Xeno™ Internal Positive Control (IPC) DNA was added in all workflows.

### 2.4. Real-Time PCR

The VetMAX™-Plus qPCR Master Mix (ThermoFisher Scientific, Waltham, MA, USA) was used for DNA amplification. The PCR amplification mixture comprised 12.5 µL 2× qPCR Master Mix, 400 nM (final concentration) forward/reverse primers, 150 nM (final concentration) probe, 1 µL VetMAX™ Xeno™ Internal Positive Control-VIC™ Assay (ThermoFisher Scientific, Waltham, MA, USA), 2 µL template DNA, and nuclease-free water to make up a total volume of 25 µL per reaction.

Detection and amplification of purified DNA by qPCR was performed on the StepOnePlus™ Real-Time PCR System running StepOne Software v2.3 system (ThermoFisher Scientific, Waltham, MA, USA). The cycling conditions recommended by the manufacturer of the PCR reagents were used.

### 2.5. Assay Optimisation

The primer and probe concentration of the genus-specific qPCR assay was optimised by testing primer/probe concentrations in triplicates for *B. melitensis* (BMR 18001) and *B. abortus* (B01988-18). For primer optimisation, a combination of four primer concentrations in the PCR reaction (800, 400, 200, and 100 nM) of both forward and reverse primers were tested, with a constant probe concentration in the PCR reaction of 250 nM. Then, the optimised primer concentration determined was used for probe optimisation by varying probe concentrations in the PCR reaction (250, 200, 150, 100, and 50 nM). The combination of primer and TaqMan^®^ probe concentration that yielded optimal assay performance (low cycle threshold (C_T_), efficient (steep) amplification slope, and low primer/probe concentration) was chosen for further experiments.

### 2.6. Analytical Validation

The analytical performance of the assay was determined by following the Stage 1 validation pathway in Chapter 1.1.2 of the OIE Manual of Diagnostic Tests and Vaccines for Aquatic Animals of the World Organisation for Animal Health [29].

The intended purpose of the assay is to (i) contribute to the eradication or elimination of brucellosis from defined populations, (ii) confirm the diagnosis of suspect or clinical cases and (iii) estimate the prevalence of infection or exposure to facilitate risk analysis (surveys, herd health status, disease control measures) in samples submitted for veterinary diagnostics.

#### 2.6.1. Efficiency

*Brucella abortus* bv. 1 (B01988-18 strain) with a bacterial cell count of 3.19 × 10^7^ cells/mL was diluted with PBS in a ten-fold dilution series from 10^−8^–10^0^ Blood, milk, and digested tissue sample matrix (180 µL) samples were spiked with 20 µL of the *B. abortus* dilution. DNA was purified from each dilution, and the PCR was performed in triplicate.

Standard curves for each run and dilution series were plotted showing C_T_-values versus log bacteria/PCR reaction. Linear regressions of the data were performed using Microsoft Excel and the slopes of the regression lines of the standard curve used to calculate the efficiency of the PCR assay (%) = (10^−1/slope^ − 1) × 100.

#### 2.6.2. Analytical Sensitivity

The last dilution that produced a positive result in all replicates, obtained from the efficiency analysis (70.89 bacteria/PCR reaction), was used to prepare a two-fold dilution series of *B. abortus* bv. 1 (B01988-18 strain) with PBS to cover the non-linear range of the assay. Blood, milk, and tissue sample matrix were spiked with *B. abortus*. DNA from each dilution was purified five times, and each purified DNA sample was tested by PCR five times (each dilution was tested 25 times in total) for all matrixes.

SPSS Statistics 25 software (IBM, New York, NY, USA) was used to perform a probit analysis to determine the 95% limit of detection (LOD).

There are three genome copies of the 16S-23S ITS region per *Brucella* sp. bacterium [21]. Results were expressed in bacterial copies, where one bacterium is equivalent to three genome copies.

#### 2.6.3. Analytical Specificity

The ITS qPCR assay was tested against a panel of Gram-positive and Gram-negative bacteria, as listed in Table 1. Bacterial strains were cultured, and a loop full of colonies was harvested and mixed well in 1 mL of PBS for all the strains in Eppendorf tubes. The mixtures were diluted 1:10 in PBS before the DNA was purified and tested by PCR, as described previously.

The *O. intermedium* bacterium was not available in our laboratory as a bacterial culture, and the DNA sequence of *O. intermedium* that encompassed the target region of the PCR assay was synthesised by IDT (Integrated DNA Technologies, Coralville, IA, USA).

#### 2.6.4. Repeatability

Data obtained from the sensitivity results were used to determine the inter-run and intra-run repeatability of the assay, using Microsoft Excel.

### 2.7. Diagnostic Performance on Field Samples

#### 2.7.1. Sample Preparation

A blind study of random positive and negative samples was obtained from the Bacteriology laboratory of the Department of Veterinary Tropical Diseases. A total of 56 aborted foetal tissue samples were used to evaluate the diagnostic performance of the assay. The samples comprised 24 foetal abomasal fluid samples and 32 tissue samples (which included aborted foetal liver, lung and placenta from bovine, caprine, and ovine). DNA from these samples were purified and tested using the ITS PCR and the BCSP31 PCR [16] assays in parallel.

#### 2.7.2. Real-Time PCR

The developed ITS PCR assay was compared to the BCSP31 PCR assay for diagnostic validation because the BCSP31 gene target is one of the most commonly used targets used for the detection of *Brucella* spp. in animals [30] and is routinely used by OIE reference laboratories. The BCSP31 PCR can be used in a real-time PCR format, and the primers and probe for the *Brucella* spp. described by Probert et al. [16] was used in our study. This assay was used on a different platform (StepOne real-time PCR machine, Applied Biosystems™, Foster City, CA, USA) and the VetMAX™-Plus qPCR Master Mix (as described in 2.4, but with 300 nM forward and reverse primer concentrations and 100 nM probe concentration in the final reaction.

#### 2.7.3. Diagnostic Sensitivity and Specificity Statistical Analysis

Diagnostic sensitivity and specificity of the PCR assay were estimated in the absence of a gold standard assay, by using a three-test one-population Bayesian latent class model that allowed for conditional dependence between two of the tests and independence of the third test [31].

Modes were obtained from published references [21,22,32,33]. For multiple references, the average of the modes was used (Table 2). For the sensitivity of the 16S-23S rDNA ITS PCR assay, the references reported on the diagnostic sensitivity in blood samples. For the diagnostic sensitivity in aborted material, the prior for this parameter was adjusted upwards. No reference could be obtained for “prevalence”, the proportion of tested aborted material positive for *Brucella* spp. submitted to a diagnostic laboratory in South Africa and expert opinion (Dr. A. Jonker, DVTD, UP) was obtained.

The model was run in OpenBUGS, version 3.2.3 rev 1012, a programme for Bayesian analysis of complex statistical models using Markov chain Monte Carlo techniques [34,35]. Two chains were used, and initial values were generated by forward sampling from the prior distribution for each parameter. The first 10,000 iterations were discarded, and the next 50,000 iterations were used for posterior inferences. The first 10,000 iterations were discarded because it was regarded as “burn-in” to check convergence. The simulation was run for a further number of iterations to obtain samples that could be used for posterior inference. Model convergence was assessed by visual inspection of the trace plots.

### 2.8. Ethics Statement

Ethics approval was obtained from the University of Pretoria Animal Ethics Committee with the project identification code (V004-19) on 29 January 2019. Section 20 approval was granted according to Act 35 of 1984 by the Directorate of Animal Health, South Africa on 8 January 2019.

## 3. Results

### 3.1. Assay Design

An in silico analysis was done on all available *Brucella* and closely related species. This included 132 *Brucella* sequences and 126 closely related species (*O. anthropi*, *O. intermedium*, *O. oryzae*, *O. otitici*, *O. ogrignonense*, *O. spp*, *S. virus*, *O pituitosum*, *C. tokpelaia*). The BLAST^®^ analysis of the 16S-23S rDNA ITS region of our selected reference returned 391 sequences. Sequences that were less than 15 bases were deleted and identical sequences were collapsed, resulting in 258 sequences.

**Figure 1:**
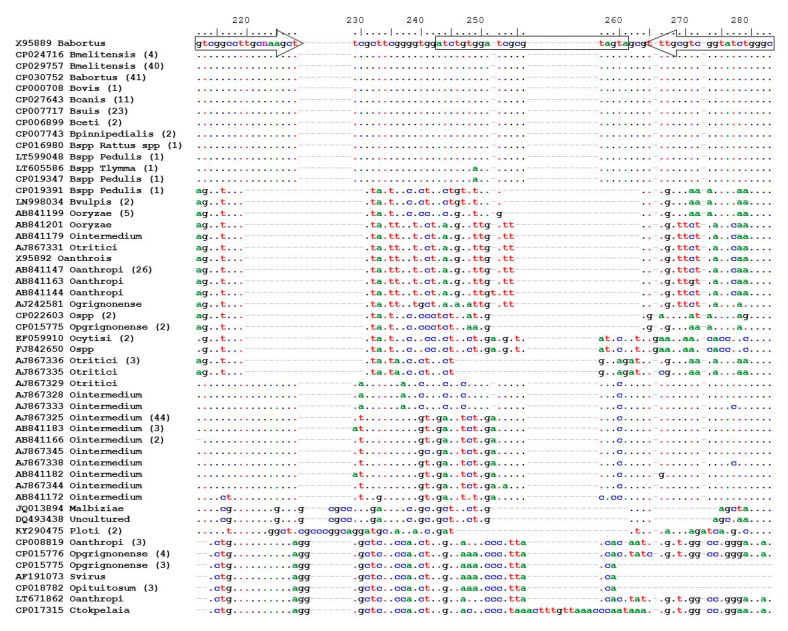
Multiple sequence alignment of the 16S-23S rDNA internal transcribed spacer (ITS) region of *Brucella* spp. and closely related *Ochrobactrum* spp. available on GenBank^®^. Primers and probe have been indicated by arrows and a rectangle respectively. X95889 was used for the nucleotide position numbering. Identical sequences have been grouped and labelled with a randomly selected sequence in the group. The number of identical sequences in a group is indicated in brackets after the name of the group.

Using the default parameters of the Primer Express software, no acceptable primer pairs were found, but when the T_m_ was increased from 60 to 61, suitable primers and probe were found. Using *Brucella abortus* X95889 for nucleotide position, primers and a hydrolysis probe were designed to specifically amplify a region from nucleotides 200–290 of all *Brucella* spp. (Figure 1 and Table 3).

A BLAST^®^ analysis of the amplicon indicated in silico specificity for *Brucella* spp. sequences.

### 3.2. Assay Optimisation

The combination of 400 nM forward and reverse primer and 150 nM probe combination resulted in an amplification plot with a low C_T_, efficient (steep) amplification slope, and low primer/probe concentration.

### 3.3. Analytical Validation

#### 3.3.1. Efficiency

**Figure 2:**
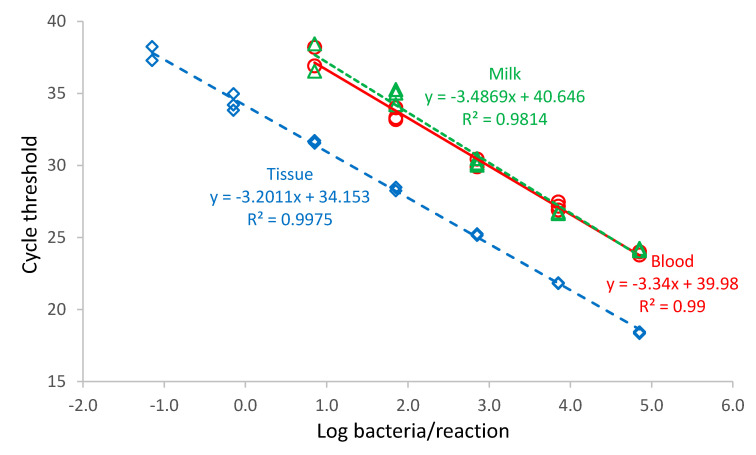
Standard curves of a genus-specific 16S-23S rDNA internal transcribed spacer *Brucella* spp. PCR assay using different matrices [i.e., blood (red circles), milk (green triangles), and tissue (blue diamonds)]. Each dilution was run in triplicate. The equation of the regression line (red line for blood, green dash for milk and blue dash for tissue) is indicated, as well as the coefficient of determination (R^2^). One bacterium contains three copies of the 16S-23S rDNA internal transcribed spacer gene.

A ten-fold serial dilution of *B*. *abortus* (10^−8^–10^0^) was prepared from a stock solution in PBS. Blood, milk, and tissue sample matrices were spiked with various dilutions of *Brucella abortus*. Amplification of the DNA was linear for positive results within the range tested, i.e., Log_10_ −0.15 to Log_10_ 4.55 bacteria/reaction (blood and milk) and Log_10_ −1.15 to Log_10_ 4.85 bacteria/reaction (tissue) (Figure 2). This was equivalent to Log_10_ 2.20 to Log_10_ 7.20 bacteria/mL blood or milk, and Log_10_ 1.20 to Log_10_ 7.20 bacteria/mL digested tissue. The efficiency of the PCR assay was 105% in tissues, 99% in blood, and 93% in milk.

#### 3.3.2. Analytical Sensitivity

**Figure 3:**
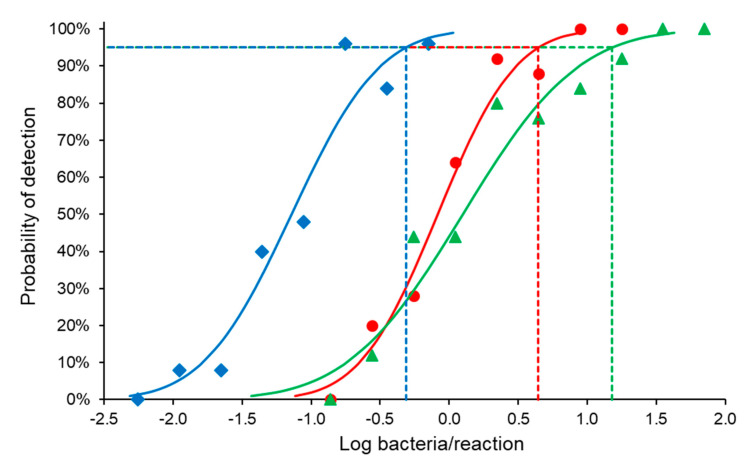
The 95% limit of detection (dotted lines) of a genus-specific 16S-23S rDNA internal transcribed spacer *Brucella* spp. PCR for tissue (diamond), blood (circle), and milk (triangle). One bacterium contains three copies of the 16S-23S rDNA internal transcribed spacer gene.

The analytical sensitivity was determined by preparing two-fold serial dilutions from Log_10_ 4.20 to Log_10_ 1.49 bacteria/reaction (blood and milk) and Log_10_ −0.15 to Log_10_ −2.85 bacteria/reaction (tissue). The 95% LOD of the assay was 4.43 *Brucella* organisms/PCR reaction in blood, 15.18 in milk, and 0.49 in tissues (Figure 3 and Table 4).

#### 3.3.3. Analytical Specificity

The developed ITS qPCR assay specifically amplified DNA from all *Brucella* spp. (*Brucella abortus* bv. 1,2 and *Brucella melitensis*) and no amplification was recorded from other closely related bacterial spp. (*Ochrobactrum anthropi*, *Ochrobactrum intermedium*, *Escherichia coli*, *Streptococcus agalactiae*, *Salmonella typhimurium*, *Pasteurella multocida*, *Enterococcus faecalis*, *Pseudomonas aeruginosa*, *Chlamydia abortus*).

#### 3.3.4. Repeatability

The standard deviation of the inter-run and intra-run reproducibility was in the range of 0.1 to 1.7. The coefficient of variation (CV) ranged from 1.7% to 4.7% (Table 5).

### 3.4. Diagnostic Performance on Field Samples

Fifty-six samples were tested by bacterial culture, BSCP and ITS qPCR (Table 6). The prevalence of brucellosis in aborted tissues was 33% (19/56). A cut-off C_T_-value of 38 (45% LOD) was used to categorise samples as negative or positive for the BSCP and ITS assays (Table 7). The cut-off C_T_ value was calculated using the equation: y = −3.2011x + 34.153, where x is the 95% confidence limit of a probability of 50% (0.22). The difference in C_T_ (ΔC_T_) observed between ITS and BCSP31 was between 7.10 and 3.24. The ITS qPCR assay produced lower C_T_-values than the BCSP31 qPCR assay.

A Bayesian latent class model was used to estimate the diagnostic sensitivity and specificity of the ITS assay as 87% and 95%, respectively. Compared to the BCSP31 assay, these values were 92% and 89% and for bacterial culture, these values were 47% and 55%, respectively (Table 8).

## 4. Discussion

We have described the development and analytical validation of a genus-specific TaqMan^®^ MGB qPCR assay to detect *Brucella* species targeting the 16S-23S rDNA ITS region. Keid et al. [22] developed a conventional ITS PCR to detect *Brucella* in various samples, including whole blood, serum, semen, and vaginal swabs, but this study was carried out exclusively in dogs. Real-time PCR has an advantage over conventional PCR because it is more sensitive and is a quicker assay to perform. Kattar et al. [21] developed an ITS qPCR assay to detect *Brucella* in humans; however, the performance of the ITS qPCR assay in detecting *Brucella* in animal specimens was not known. This study filled the gap by adding the advantage of detecting *Brucella* species in easily accessible samples (milk, blood, and tissues) for early detection, which is a more sensitive qPCR ITS assay as compared to Kattar et al. (2007), and lastly, this assay has been optimised in animal samples.

Prior to design of the assay, an in silico analysis was carried out to determine conserved region of the 16S-23S rDNA ITS region of all *Brucella* species as well as closely related bacteria, using the BLAST^®^ analysis on GenBank.

The BLAST^®^ tool showed in silico specificity of the assay. In the laboratory, specificity was determined by testing *Brucella* species against a panel of closely related bacteria. The developed ITS PCR assay showed good specificity by amplifying DNA from all *Brucella* species and not amplifying closely related bacterial species, such as *Ochrobactrum anthropi* and *O. intermedium*. Our results were in agreement with Keid et al. [22]; their ITS-PCR was specific, since there was no amplification of *O. anthropi* DNA; however, this was a conventional ITS PCR. The same result was obtained by Kattar et al. [21], who used hybridisation probes specific to the ribosomal 16S–23S ITS region, *omp*31 and *omp*25. All three assays exhibited an analytical sensitivity and specificity of 100%.

The developed ITS assay efficiency was 99% in blood, 93% in milk, and 105% in tissues. An efficiency exceeding 100% was recorded in tissues. This may be a result of the digestion method used to purify DNA from tissues, which differed from the method used for liquid samples by an additional incubation step of the tissue sample in proteinase kinase before purification of the nucleic acid. The digestion of tissue and *Brucella*, a difficult organism to lyse, with PK solution enhances lysing of the bacteria [37]. This may have caused the higher efficiency value in tissues compared to blood and milk.

The 95% LOD of the developed ITS-PCR assay was 4.43, 15.18, and 0.49 *Brucella* organisms/PCR reaction in blood, milk, and tissues respectively of *B. abortus* bv. 1 (B01988-18 strain), which demonstrated the sensitivity of the assay. This is of diagnostic importance, because latent asymptomatic animals serve as a source of infection to other animals (maintaining the disease), as well as humans [12]. In humans, brucellosis can progress to sub-clinical or chronic stages if not detected early and treated promptly [38]. 

The increased sensitivity of the assay in tissue samples may be a result of the different nucleic acid purification protocol used for tissue, compared to liquid samples. The tissue protocol included an additional proteinase kinase digestion step, which may have increased the intra-cellular release of the bacteria, allowing for the more sensitive detection of *Brucella* organisms. Investigation into the addition of a digestion step for liquid samples when purifying nucleic acid, to increase the sensitivity of detection of *Brucella* organisms is warranted.

The standard deviation of the inter-run and intra-run reproducibility was in the range of 0.1 to 1.7. The CV ranged from 1.7% to 4.7%. Minimal variation in results was demonstrated.

A blind test was carried out on 56 samples that were obtained from the bacteriology laboratory (DVTD, UP), to determine this assay’s potential as a screening tool in an abortion panel. Nucleic acid was purified and amplified by ITS and BCSP31 qPCR assays. Then, test results were compared to culture results. Samples that were positive on ITS but negative on both BCSP31 and culture were retested by bacterial culture. It was interesting to note that when these samples were re-cultured, there was growth of *Brucella* colonies, which were initially negative on culture. This demonstrated that *Brucella* cases may be “missed” with traditional diagnostic techniques. It also confirms that brucellosis is under-reported in the country, since bacterial culture isolation is the “gold standard” of identification. The initial negative results obtained from culture could be as a result of the use of selective media. Farrell’s selective media is used mostly for the isolation of the smooth *Brucella*, and this media inhibits the growth of *B.* ovis as well as some *B. melitensis* and *B. abortus* strains [39]. It may be postulated that these tissue samples could be infected with any of these three *Brucella* species and supports the assertion that the developed ITS assay can detect any species of *Brucella*.

We compared the diagnostic sensitivity and specificity of the ITS and BCSP31 qPCR assays in aborted tissue samples. The two assays were performed on the same PCR platform to provide a direct comparison between the two assays, but the PCR platform used differed from the platform published for the BCSP31 assay. To the best of our knowledge, there is no publication on the validation of an ITS qPCR assay to detect *Brucella* spp. in aborted tissue samples. Out of the 56 samples tested, 9 were positive with all three tests, 37 were negative with all tests, 7 were negative with both bacterial culture and the BCSP31 assay but positive on the ITS assay: this could be a result of small amounts of target DNA in these samples. One sample was positive with both the ITS and BCSP31 assays but negative on bacterial culture. This may indicate the presence of dead organisms in this tissue sample. Two samples were positive with both bacterial culture and the ITS assay but negative with the BCSP31 assay. This could be explained because of the low number of bacteria in this sample. The C_T_ values of the ITS-PCR for these samples were 37.19 and 36.42, which indicated that the ITS assay could detect smaller amounts of DNA in a sample [22] compared to the BCSP31. It is worth noting that BCSP31 is highly conserved in all *Brucella* species and biovars, except for *Brucella ovis* [40]. It may be that this sample could be *Brucella ovis* and supports the assertion that the developed ITS assay can detect any species of *Brucella*.

The ITS qPCR assay produced lower C_T_ values than the BCSP31 assay, suggesting that the ITS assay is more sensitive than the BCSP31 assay. The difference in C_T_ values observed between these assays could be a result of the DNA copy number in these gene targets. The BCSP31 gene, which encodes for a 31-kDa *B. abortus* antigen, is present as a single copy, whereas the 16S-23SrDNA ITS region exists as three copies [21], and it is likely that a gene target with multiple copy numbers is more sensitive than a single copy gene.

The difference in C_T_ (ΔC_T_) observed between the ITS and BCSP31 assays was between 7.10 in aborted foetal tissues and 3.24 in abomasal fluid. This result agrees with those of Katter et al. [21], who found out that the C_T_ values were three cycles lower for ITS-PCR in tissues than that of blood. This indicated a higher bacterial load in tissues and the possible use of these assays for diagnosing the disease. 

The diagnostic sensitivity and specificity of the ITS and BSCP31-qPCR was 87%/95% and 92%/89% respectively, and for culture, it was 47% and 55% respectively, which indicated that PCR is more sensitive than culture. Our ITS assay showed a greater specificity of 95% than the BCSP31 assay, with a specificity of 89%. In contrast, Katter et al. [21] recorded the sensitivity and specificity of an ITS qPCR assay as 66.7% and 99.7% respectively, compared to culture with 77% and 100% for whole blood and paraffin-embedded tissue. Keid et al. [22] standardised and evaluated an ITS PCR on whole blood of naturally infected dogs for the detection of *Brucella* species: dogs were assigned to infected and non-infected groups based on culture and rapid slide agglutination tests, and a sensitivity and specificity of 100% was reported. The disparity in diagnostic sensitivity and specificity of PCR from various studies could be due to the use of different protocols for DNA extraction, amplification, different target genes and the choice of reference test [22,41].

Interestingly, the ITS assay performance, when statistically compared to BCSP31 in our study, does not reflect the theory of ‘‘an increased sensitivity corresponding to a multiple rDNA copy number’’. This could be as a result of the prior values used in determining the sensitivity of the ITS assay in the Bayesian statistical model. The priors were obtained from the prevalence, sensitivity, and specificity of the results from published literature. There is lack of information available for studies carried out on the detection of *Brucella* in aborted foetal tissues using the BCSP31 gene target by qPCR. Hence, priors for the BCSP31 assay was obtained from Richtzenhain et al. [32], which is a multiplex conventional PCR. This might have contributed to the difference in sensitivity in our result. According to Ling et al., non-identifiable latent class models are known to be greatly influenced by the subjective prior information used [42].

In addition to the BCSP31 assay, an insertion sequence 711 (IS711)-based real-time PCR has been used to detect *Brucella* spp. in blood and tissue samples in wild boars [43]. The advantages of this assay are that IS711 is present in multiple copies in the Brucella genome and represents a stable genetic element with regard to the number and position in the genomes of various Brucella species [8,44]. Most species contain at least one copy of the IS element at a unique chromosomal location [45]. The AMOS-PCR is a locus-specific multiplexing assay to differentiate Brucella species and is based on IS711 [46].

Loop-mediated isothermal amplification (LAMP) is one of the most widely used isothermal amplification assays used for the detection of several bacterial pathogens including *Brucella* sp. in clinical samples [47,48,49,50]. Since LAMP requires multiple primers, it involves rigorous optimisation and increases the chance of leftover contamination, giving false-positive results in many instances [51]. The LAMP technique is simple because the whole process of amplification and detection is done in a single step in which the reaction components are subjected to isothermal conditions [52]. This technique can be used on the field. It also has the advantage of working at constant temperature, results can be viewed directly without post-amplification protocols [53].

The prevalence of brucellosis in aborted foetal tissues was 33% (19/56). This shows that prevalence has increased from initial diagnostic reports. Kolo et al. [54] reported a prevalence of 12.5% (25/200) from screened cattle tissues by conventional ITS-PCR; however, this was conducted on abattoir samples from apparently healthy animals. In a study carried out in Egypt, a total of 47 aborted foetuses with related placenta were examined. *Brucella* spp. DNA was detected in 25.5% (12/47)) of tissue samples by conventional PCR [55]. 

About 41.5% of the nation’s herds were tested between 1977 and 1978. A prevalence of 6.6% was recorded. Northeastern parts of Kwa-Zulu-Natal [56] reported a prevalence of 1.45–15.6%.

Another issue of concern is that because the current bovine brucellosis testing scheme is compulsory only for high-risk herds and voluntary for all other herds and livestock owners [57], the status of numerous herds that were not classified as high-risk remains unknown [57]. This leads to the assumption that the prevalence of the disease in the country is higher than previously reported [58]. There is minimal information on brucellosis in small ruminants, pigs, and wildlife [10].

The presence of *Brucella* spp. in livestock is a major risk to public health, because livestock and animal products are the only source of infections in humans [59].

## 5. Conclusions

The diagnosis of brucellosis mainly relies on serological and bacteriological techniques. These methods reach only suitable levels of sensitivity at a late phase of the disease when clinical signs are already apparent. Therefore, early and correct diagnosis of the disease by our assay aids in the control of the disease in South Africa. The developed assay is a valuable tool in the early detection of the *Brucella* pathogen.

## Figures and Tables

**Table 1:** Bacterial strains used in this study.

Organism	Strain
*Brucella abortus* bv. 1	B01988-18
*Brucella abortus* bv. 1	B01897-18
*Brucella abortus* bv. 2	B01872-18
*Brucella abortus* bv. 1	B01853-18
*Brucella abortus* bv. 1	B01060-18
*Brucella abortus* bv. 1	B01100-18
*Brucella melitensis*	BMR 1 8001
*Ochrobactrum anthropi*	ATCC 49,687
*Ochrobactrum intermedium*	DNA (Genbank accession number AJ867325) *
*Escherichia coli*	ATCC 25,922
*Streptococcus agalactiae*	ATCC 27,956
*Salmonella typhimurium*	ATCC 13,311
*Pasteurella multocida*	ATCC 12945/43137
*Enterococcus faecalis*	ATTC 29,212
*Pseudomonas aeruginosa*	ATCC 27853
*Chlamydia abortus*	DVTD, UP

ATCC—American Type Culture Collection, bv—biovar, DVTD, UP—Department of Veterinary Tropical Diseases, University of Pretoria. * DNA synthesised by Integrated DNA Technologies (USA).

**Table 2:** Priors used in a three-test one-population Bayesian latent class model for the diagnosis of *Brucella* spp. in field samples by bacterial culture, *Brucella* cell surface protein polymerase chain reaction: BCSP31 PCR assay and ITS PCR assay. Prevalence = proportion of tested aborted material positive for *Brucella* spp.

Parameter	Mode	5/95th Percentile	α-Value	β-Value
Sensitivity_Culture	0.440	0.10	1.6376	1.8114
Specificity_Culture	0.590	0.15	1.9571	1.6651
Sensitivity_BCSP	0.990	0.70	8.7838	1.0786
Specificity_BCSP	0.930	0.70	12.1696	1.8407
Sensitivity_ITS	0.900	0.70	15.0342	2.5594
Specificity_ITS	0.998	0.80	13.6318	1.0253
Prevalence	0.125	0.80	1.1386	1.9702

**Table 3:** Primers and TaqMan^®^ minor groove binder (MGB) probe targeting the 16S-23S internal transcribed spacer (ITS) region of *Brucella* spp. and labelled with 6-caboxyfluorescein (6-FAM) dye, nt—nucleotide, T_m_—melting temperature. The length of the amplicon was 72 base pairs.

Name	Sequence (5′→3′)	Length (nt)	T_m_	%GC
BrucellaITS_F	GTCGGCCTTGCGAAGCT	17	59	65
BrucellaITS_R	GCCCAGATACCGACGCAAA	19	61	58
BrucellaITS_P	FAM-ATCTGTGGATCGCGTAGTA-MGB	19	69	47

**Table 4:** The 95% limit of detection of a genus-specific 16S-23S rDNA internal transcribed spacer (ITS) *Brucella* spp. PCR determined by probit analysis. CI—confidence interval. One bacterium is equivalent to three genome copies.

Sample	Bacteria/PCR Reaction	Genome Copies/PCR Reaction	Log Bacteria/PCR Reaction	Lower 95% CI	Upper 95% CI
Blood	4.43	13.30	0.65	0.48	0.89
Milk	15.18	45.54	1.18	0.97	1.49
Tissue	0.49	1.45	−0.31	−0.50	−0.05

**Table 5:** Inter- and intra-assay reproducibility for the detection of *Brucella* spp., SD—standard deviation, C_T_—cycle threshold, CV—coefficient of variation.

Sample	Log bacteria/PCR Reaction	Inter-run SD	Intra-run SD	Total Mean C_T_	Total SD	CV (%)
Blood	1.25	0.13	0.56	31.35	0.56	1.77
Blood	0.95	0.35	0.81	32.79	0.88	2.68
Blood	0.65	0.32	0.8	33.91	0.82	2.42
Blood	0.35	0.25	0.92	35.07	0.94	2.68
Blood	0.04	0.8	0.87	36.06	1.13	3.14
Blood	−0.26	0.58	0.94	36.29	1.04	2.86
Blood	−0.56	0.41	1.19	36.71	1.17	3.18
Milk	1.85	0.32	0.81	31.00	0.82	2.65
Milk	1.55	0.31	0.82	32.74	0.80	2.45
Milk	1.25	0.43	1.22	33.73	1.37	4.07
Milk	0.95	0.46	1.01	34.23	1.14	3.34
Milk	0.65	0.32	0.90	35.41	1.06	2.98
Milk	0.35	0.40	0.84	36.03	0.91	2.51
Milk	0.04	0.29	0.60	36.76	0.54	1.46
Tissue	−0.15	0.27	1.74	34.87	1.61	4.61
Tissue	−0.45	0.52	1.32	35.78	1.30	3.62
Tissue	−0.75	0.43	1.47	34.67	1.44	4.16
Tissue	−1.05	1.05	0.00	36.16	1.51	4.17

**Table 6:** Comparison of bacterial culture, BCSP31 and ITS qPCR assays for the diagnosis of *Brucella* spp. from foetal abomasal fluid and foetal tissue samples. Cut-off values of + < cycle threshold [36] = 38.00 > − were used to categorise positive (+) and negative (−) samples for both the BCSP31 and ITS (45% limit of detection (LOD)) qPCR assays. ΔC_T_ = C_T_ (ITS) − C_T_ (BCSP31).

Sample Name	Sample Type	Culture	BCSP	ITS	ΔC_T_
S0713/19	Foetal abomasal fluid	+	+(20.02)	+(16.78)	3.24
B1644-19_S5151-19	Foetal abomasal fluid	+	+(20.60)	+(17.25)	3.35
B705/19_S91b/19	Foetal abomasal fluid	+	+(21.53)	+(18.16)	3.37
B1988-18	Foetal abomasal fluid	+	+(26.21)	+(22.40)	3.81
B1631_18	Foetal abomasal fluid	-	-	+(37.95)	
B1756/19_S2327/19	Foetal abomasal fluid	-	-	−(39.89)	
5699/18	Foetal abomasal fluid	-	-	-	
7683/18	Foetal abomasal fluid	-	-	-	
B1167_19	Foetal abomasal fluid	-	-	-	
B1377/18	Foetal abomasal fluid	-	-	-	
B1431/19	Foetal abomasal fluid	-	-	-	
B1499_18	Foetal abomasal fluid	-	-	-	
B1554/19	Foetal abomasal fluid	-	-	-	
B1568_19	Foetal abomasal fluid	-	-	-	
B1588_19	Foetal abomasal fluid	-	-	-	
B1593/19_B632/18	Foetal abomasal fluid	-	-	-	
B1800/18_S290/18	Foetal abomasal fluid	-	-	-	
B1803/19_S238/19	Foetal abomasal fluid	-	-	-	
B2063/19_S2718/19	Foetal abomasal fluid	-	-	-	
B618/18_B661/18	Foetal abomasal fluid	-	-	-	
B646/19_S865/19	Foetal abomasal fluid	-	-	-	
B647/19_S865/19	Foetal abomasal fluid	-	-	-	
B7482/19	Foetal abomasal fluid	-	-	-	
S3855_18	Foetal abomasal fluid	-	-	-	
B1644/19/S2151/19	Aborted foetal tissue	+	+(27.34)	+(20.24)	7.10
B1857/19_S2461/19	Aborted foetal tissue (bovine lung)	+	+(27.83)	+(21.65)	6.18
B706/19	Aborted foetal tissue	+	+(28.76)	+(21.90)	6.86
B705/19/591b/19	Aborted foetal tissue	+	+(29.56)	+(23.67)	5.89
B1857/19_S2461/19	Aborted foetal tissue (bovine liver)	+	+(36.23)	+(31.02)	5.21
B1569_19_RB	Aborted foetal tissue	+	−(39.58)	+(35.44)	4.14
B1569_19_LN	Aborted foetal tissue	+	-	+(37.19)	
B1144_17	Aborted foetal tissue	-	+(36.51)	+(31.90)	4.61
B1860/19_S2445/19	Aborted foetal tissue (bovine lung)	-	-	+(29.26)	
B2063/19_S2718/19	Aborted foetal tissue (bovine liver)	-	-	+(34.92)	
B215/18_S504/18	Aborted foetal tissue (ovine liver)	-	-	+(35.68)	
B2063/19_S2718/19	Aborted foetal tissue (bovine lung)	-	-	+(36.80)	
B1756/19_S2327/19	Aborted foetal tissue (caprine placenta)	-	-	+(37.59)	
B2033/19_S2672/19	Aborted foetal tissue (ovine lung)	-	-	+(37.92)	
B2033/19_S2672/19	Aborted foetal tissue (ovine placenta)	-	-	−(38.08)	
B1010/19_S1309/19	Aborted foetal tissue (ovine lung)	-	-	−(38.12)	
B1010/19_S1309/19	Aborted foetal tissue (ovine placenta)	-	-	−(38.69)	
B1488/18_B1434/18	Aborted foetal tissue (ovine placenta)	-	-	−(39.24)	
B1010/19_S1309/19	Aborted foetal tissue (ovine liver)	-	-	−(39.79)	
B1593/18_B632/18	Aborted foetal tissue (ovine lung)	-	-	-	
B1702_19_S2207_19	Aborted foetal tissue	-	-	-	
B1702_19_S2267_19	Aborted foetal tissue	-	-	-	
B1800/18_S290/18	Aborted foetal tissue (ovine lung)	-	-	-	
B1800/18_S290/18	Aborted foetal tissue (ovine placenta)	-	-	-	
B2033/19_S2672/19	Aborted foetal tissue (ovine liver)	-	-	-	
B215/18_S504/18	Aborted foetal tissue (ovine lung)	-	-	-	
B540/18_S631/19	Aborted foetal tissue (caprine liver)	-	-	-	
B540/19_S631/19	Aborted foetal tissue (caprine lung)	-	-	-	
B647/19_S8651/19	Aborted foetal tissue (ovine placenta)	-	-	-	
B771/18_S1322/18	Aborted foetal tissue (caprine placenta)	-	-	-	
B856/18_S1430/18	Aborted foetal tissue (caprine lung)	-	-	-	
B856/18_S1430/18	Aborted foetal tissue (caprine liver)	-	-	-	

**Table 7:** Fifty-six aborted foetal samples submitted to DVTD for *Brucella* spp. testing and classified according to test results. BSCP31—PCR assay [16], Culture—bacterial blood agar culture, ITS—internal transcribed spaced PCR (this study). The header refers to the results for culture and the BSCP31 assay.

	Culture+	Culture+	Culture-	Culture-
	BSCP31+	BSCP31-	BSCP31+	BSCP31-
**ITS+**	9	2	1	7
**ITS-**	0	0	0	37

**Table 8:** Estimates of the diagnostic sensitivity and specificity of the PCR assay.

	Median	95% Probability Interval
Sensitivity of the ITS-PCR	0.868	0.701–0.963
Specificity of the ITS-PCR	0.949	0.800–0.996
Sensitivity of the BCSP31-PCR	0.917	0.700–0.993
Specificity of the BCSP31-PCR	0.886	0.701–0.977
Sensitivity of bacterial isolation	0.469	0.102–0.868
Specificity of bacterial isolation	0.549	0.150–0.903
